# The association of early life factors with depression and anxiety in adults aged 40–69 years: a population-based cohort study

**DOI:** 10.1038/s41398-024-03006-7

**Published:** 2024-07-20

**Authors:** Ruirui Wang, Mengyao Shi, Qilu Zhang, Jing Zhang, Lulu Sun, Yiming Jia, Zhengbao Zhu, Tan Xu, Yonghong Zhang

**Affiliations:** Department of Epidemiology, School of Public Health and Jiangsu Key Laboratory of Preventive and Translational Medicine for Geriatric Diseases, Suzhou Medical College of Soochow University, Suzhou, China

**Keywords:** Psychology, Diseases

## Abstract

This study was aimed to explore the longitudinal association of five early life factors (breastfeeding, maternal smoking around birth, birth weight, being born in a multiple birth, and adoption) during the in-utero, perinatal, and early childhood development stages with incidence of depression and anxiety in adults aged 40–69 years. We used data from the UK biobank, 5,02,394 participants aged 40–69 years were recruited between 2006 and 2010. Participants provided information on early life exposures through touchscreen questionnaires or verbal interviews at baseline. The primary outcomes, depression, and anxiety, were defined according to the International Classification of Diseases, 10th Revision. Hazard ratios (HR) and 95% confidence intervals (CI) for each factor were reported. During a median follow-up of 13.6 years, 16,502 (3.55%) participants developed depression, and 15,507 (3.33%) developed anxiety. After adjusting for potential confounders, increased risk of depression was found to be significantly associated with non-breastfeeding (HR, 1.08; 95% CI, 1.04–1.13), maternal smoking around birth (HR, 1.19; 95% CI, 1.14–1.23), being born in multiple births (HR, 1.16; 95% CI, 1.05–1.27), low birth weight (HR, 1.14; 95% CI, 1.07–1.22), and being an adoptee (HR, 1.42; 95% CI, 1.28–1.58). Increased risk of anxiety was associated with non-breastfeeding (HR, 1.09; 95% CI, 1.04–1.13), maternal smoking around birth (HR, 1.11; 95% CI, 1.07–1.16), being born in a multiple births (HR, 1.05; 95% CI, 0.95–1.17), low birth weight (HR, 1.12; 95% CI, 1.05–1.20), and being an adoptee (HR, 1.25; 95% CI, 1.10–1.41). Each of these five early life factors can be considered as early life risk factors for incident depression and anxiety in adulthood independently. The dose-response relationship was also observed, suggesting that with an increase in the number of early life risk factors, the likelihood of experiencing depression and anxiety also increased. These findings highlighted the imperative consideration of early life factors in comprehending the susceptibility to mental health disorders later in life, including non-breastfeeding, maternal smoking around birth, being born in multiple births, low birth weight, and being an adoptee.

## Introduction

Depression and anxiety, affecting more than 300 million individuals worldwide, stand as formidable challenges to global mental health [1]. These conditions not only impair daily functioning but also escalate the risk of chronic physical disorders, encompassing obesity, diabetes, cognitive impairment, cancer, cardiovascular diseases, and premature mortality [2–5]. While existing research has extensively explored various factors contributing to these mental health conditions, a comprehensive understanding of their development remains elusive.

Previous studies on risk factors explored the associations of depression and anxiety with stressors, specific sociodemographic factors (e.g., being female), social disadvantages, income inequality, poor lifestyle habits, environmental pollutants, and parental depression [5–14]. Additionally, adverse childhood experiences, including maltreatment, cannabis consumption, and unfavorable living conditions during critical early life stages, have been shown were linked to an individual’s vulnerability to subsequent depressive episodes [15–19]. Moreover, several studies also identified intrauterine and perinatal factors, such as maternal smoking during pregnancy, birth weight, gestational age, fetal position, mode of delivery, and breastfeeding during infancy, were associated with the risk of subsequent neuro-psychiatric disorders [20–27].

These findings emphasize the crucial role played by early life factors in shaping the course of depression or anxiety, further research was warranted to investigate the long-term impact of these early life determinants on mental health in adult populations. There is a notable absence of a systematic, prospective study featuring a sizable sample size to thoroughly examine the relationship between early life factors and the occurrence of depression and anxiety in adulthood, particularly in middle-aged and older adults. This prospective cohort study, utilizing data from the UK Biobank, was designed to investigate the association between five early life factors (breastfeeding, maternal smoking around birth, birth weight, being born in a multiple birth, and being an adoptee) in utero, perinatal, and early childhood developmental stages and the incidence of depression and anxiety in adults aged 40–69 years.

## Methods

### Study population

The UK Biobank study design and methods have been reported in detail previously [28]. To summarize briefly, the UK Biobank is a very large and highly detailed prospective study involving over 5,00,000 participants aged 40–69 years who were recruited between 2006 and 2010. This study included data obtained from a touchscreen questionnaire, physical measurements, sample assays, and longitudinal follow-ups on a wide range of health-related outcomes. From 2016 to 2017, 1,57,366 participants were invited to complete online follow-up questionnaires that focused on information about diet, mental health, cognitive function, etc. [29]. The North West Multi-Centre Ethics Committee granted ethical approval to the UK Biobank (reference: 16/NW/0274), and all participants provided written informed consent prior to their participation in the study. In the present study, participants with valid data on early life factors at baseline were included, and individuals with depression or anxiety at baseline (self-reported or diagnosed by the International Classification of Diseases 10th Revision, ICD-10) were excluded. More details about the participant selection are shown in eFigure 1 in the Supplement.

### Exposure assessment and covariates

Information on early life exposure for participants was collected through touchscreen questionnaires or verbal interviews at baseline. Information on breastfeeding was obtained using the question “Were you breastfed when you were a baby?” The response options presented were “Yes,” “No,” “Do not know,” and “Prefer not to answer.”. Information on maternal smoking around birth was ascertained using the question “Did your mother smoke regularly around the time when you were born?”. The response options presented were “Yes,” “No,” “Do not know,” and “Prefer not to answer.”. Multiple birth information was obtained using the question “Are you a twin, triplet, or other multiple birth?”. The response options presented were “Yes,” “No,” “Do not know,” and “Prefer not to answer.”. Information on birth weight was collected through verbal interviews in which participants were asked to enter their birth weight in person. Information on adoption was collected via touchscreen questionnaires using the question, “Were you adopted as a child?” The response options presented were “Yes,” “No,” “Do not know,” and “Prefer not to answer.” Responses of “Prefer not to answer” and “Do not know” were set as “missing” values in the subsequent analysis. Early life risk factors were defined as non-breastfeeding, maternal smoking around birth, being born in multiple births, low birth weight (<2.5 kg) [30], and being an adoptee (adopted as a child).

Sociodemographic factors, including age, sex, and ethnicity were self-reported at the baseline assessment stage. Ethnic background was classified as White, Black, Asian, or mixed/other. The Townsend Deprivation Index used as an indicator of socioeconomic status, was derived from the current residential postcode using census data on unemployment, non-car ownership, non-home ownership, and household overcrowding. Higher scores indicated a higher degree of deprivation [31]. Participants reported their educational qualifications as holding a college or university degree, A levels/AS levels or equivalent, O levels/General Certificate of Secondary Education (GCSE) or equivalent, Certificate of Secondary Education (CSE) or equivalent, National Vocational Qualification (NVQ), Higher National Diploma (HND), Higher National Certificate (HNC) or equivalent, other professional qualifications (e.g., nursing, teaching), and none of the above (equivalent to a qualification lower than a high school diploma) [31]. Current average total household income before tax was self-reported as being <£18,000, £18,000–£30,999, £31,000–£51,999, £52,000–£1,00,000 and >£1,00,000. Current smoking status was self-reported and categorized as never, former, or current smoker. Current alcohol consumption status was categorized as never, former, or current drinker. Trained nurses measured participants’ weight and height; body mass index was calculated as participants’ weight in kilograms divided by the square of their height in meters. Current physical activity was calculated as the sum of the metabolic equivalent of task (MET) minutes/week for moderate activity plus two instances of vigorous activity, expressed as metabolic equivalents (MET-min/week). Low physical activity was defined as MET minutes per week <150 min/week [32].

### Outcome assessment

The primary outcomes of this study were symptoms of depression and anxiety recorded during follow-up, and defined according to the ICD-10. Incident depression was defined using blocks F30-F39, incident anxiety was defined using blocks F40-F41. During the follow-up, the time of death was recorded for deceased participants, the time of loss was recorded for participants who failed to participate in the follow-up, and all remaining cases were followed up until the latest review point.

Depression and anxiety scale scores were measured using the Patient Health Questionnaire-9 (PHQ-9) and the Generalized Anxiety Disorder-7 Questionnaire (GAD-7), which served as secondary outcomes. The PHQ-9 is a depression screening instrument containing nine questions listed on a four-point ordinal scale ranging from 0 to 3. The total score ranges from 0 to 27, with higher scores indicating greater symptom severity [33]. A score of 10 or higher indicated the presence of current depressive symptoms and was used to create a binary variable for depression outcomes. Depression severity can be classified as mild (a score of 10–14), moderate (a score of 15–19), and severe (a score of 20–27) [34]. The GAD-7 is a 7-item anxiety scale that uses the same four-point ordinal scale as the PHQ-9. The total score ranges from 0 to 21, with a higher score indicating more severe anxiety. A score of 10 or higher indicating the presence of current anxiety symptoms, and this threshold was subsequently used to create a binary variable for anxiety outcomes. Anxiety severity can be classified as mild (a score of 10–13), moderate (a score of 14–18), and severe (a score of 19–21) [35, 36]. The details of the PHQ-9 and GAD-7 scales are shown in eTables 1–2 in the Supplement.

### Statistical analysis

The baseline characteristics of the study population were described as means with standard deviations for continuous variables and frequencies with percentages for categorical variables. Differences in baseline characteristics were compared using Student’s *t*-tests for continuous variables and chi-square tests for categorical variables. Cox proportional hazards models were used to investigate the associations between each early life risk factor and the risk of incident depression and anxiety as defined by the ICD-10, using the follow-up time as the timescale variable. The results are expressed as hazard ratios (HRs) and their 95% confidence intervals (CIs). Important covariates were selected as confounders and adjusted for in the multivariable analysis. Model 1 was not adjusted for any covariates. Model 2 was adjusted for age (continuous), sex (categorical), ethnicity (categorical), Townsend Deprivation Index (continuous), education level (categorical), current household income (categorical), current smoking status (categorical), current alcohol consumption status (categorical), body mass index (continuous), current physical activity (categorical), and family history of depression (categorical).

The complexity and correlation of early life factors could not be ignored, and further interaction tests were performed on early life risk factors and incident depression and anxiety, using the likelihood ratio test to compare models with or without a cross-product term. The association between the number of early life risk factors and the risk of depression and anxiety were analyzed. Simple scores were easier to interpret and apply in practice, whereas the weighted score, which considered the effect value of each factor on the outcomes, was more statistically reasonable. Therefore, we also examined the association of weighted early life risk scores with depression and anxiety (eMethods in the Supplement).

In the analyses of secondary outcomes, the distribution of depression and anxiety severity as defined by the PHQ-9 and GAD-7 according to each early life risk factor status was analyzed using a chi-square test. Multivariable logistic regression models were used to estimate the odds ratio (OR) and 95% CI for the association of each early life risk factor with both depression (PHQ-9 ≥ 10 score) and anxiety (GAD-7 ≥ 10 score). The models were adjusted in the same covariables as for the primary outcome.

Multiple imputation was used to fill in missing covariate data using the PROC MI procedure in SAS software. All statistical analyses were performed using SAS version 9.4 (SAS Institute Inc., Cary, NC, USA). All *P* values were two-sided, and *P* < 0.05 was considered statistically significant.

## Results

### Baseline characteristics

A comparison of baseline characteristics between the populations included in the main and secondary analyses is shown in eTable 3 in the Supplement. The baseline characteristics of the population included in the main analysis, according to participants with or without ICD-10 classified depression and anxiety, are presented in Table 1. Individuals with depression or anxiety were more likely to be female and current smokers. They also had a higher Townsend Deprivation Index and body mass index; a lower proportion of college degrees; a higher proportion of low income, lower of physical activity, and a higher proportion of family history of depression.

**Table 1:** Baseline characteristics for the participants included in this study.

Baseline characteristics	Depression		Anxiety	
	Yes	No	Yes	No
Number of participants	16,502	4,48,491	15,507	4,49,486
Age, years	56.86 ± 8.27	56.59 ± 8.10	57.73 ± 8.10	56.56 ± 8.11
Male, %	6329 (38.35)	2,09,524 (46.72)	5261 (33.93)	2,10,592 (46.85)
White, %	15,631 (94.72)	4,23,555 (94.44)	14,762 (95.20)	4,24,424 (94.42)
Townsend Deprivation Index	−0.53 ± 3.39	−1.38 ± 3.05	−0.86 ± 3.29	−1.37 ± 3.05
University or college degree, %	3896 (23.61)	1,49,289 (33.29)	3846 (24.80)	1,49,339 (33.22)
Low income, %	5854 (35.47)	99,548 (22.20)	5209 (33.59)	1,00,193 (22.29)
Current cigarette smoking, %	2846 (17.25)	44,086 (9.83)	2140 (13.80)	44,792 (9.97)
Current alcohol drinking, %	14,467 (87.67)	4,14,830 (92.49)	13,765 (88.77)	4,15,532 (92.45)
Low physical activity, %	7190 (43.57)	1,56,970 (35.00)	6539 (42.17)	1,57,621 (35.07)
Body mass index, kg/m^2^	28.56 ± 5.61	27.32 ± 4.69	27.92 ± 5.28	27.34 ± 4.71
Family history of depression, %	2219 (13.45)	35,755 (7.97)	1926 (12.42)	36,048 (8.02)
Early life factor, %				
Breastfed as a baby	8499 (69.88)	2,49,878 (72.84)	8062 (70.69)	2,50,315 (72.80)
Maternal smoking around birth	4783 (34.43)	1,11,192 (28.73)	4233 (32.15)	1,11,742 (28.82)
Part of a multiple birth	429 (2.67)	9859 (2.24)	369 (2.44)	9919 (2.25)
Birth weight				
Normal (2.5–4 kg)	6483 (73.60)	1,89,433 (76.60)	6180 (74.19)	1,89,736 (76.58)
High (>4 kg)	1229 (13.95)	33,198 (13.42)	1112 (13.35)	33,315 (13.45)
Low (<2.5 kg)	1096 (12.44)	24,662 (9.97)	1038 (12.46)	24,720 (9.98)
Adopted as a child	346 (2.11)	6310 (1.41)	278 (1.80)	6378 (1.43)

Continuous variables are expressed as mean ± standard deviation; categorical variables are expressed as number (percentage).

### Association of early life factors with depression and anxiety coded by ICD-10

After excluding individuals with known depression or anxiety at baseline, with a median follow-up of 13.6 years, 16,502 (3.55%) participants developed depression, and 15,507 (3.33%) participants developed anxiety. The mean (standard deviation) age at first diagnosis was 56.86 (8.27) years for depression and 57.73 (8.10) years for anxiety.

The associations of each early life risk factor with depression and anxiety are shown in Table 2. After adjusting for potential confounders in Model 2, all five early life risk factors were associated with an increased risk of depression and anxiety. Compared with participants who underwent breastfeeding in infancy, the multivariate-adjusted HRs (95% CIs) for those without breastfeeding in infancy were 1.08 (1.04, 1.13) for depression and 1.09 (1.04, 1.13) for anxiety. Compared with participants who did not experience maternal smoking around birth, the multivariate-adjusted HRs (95% CIs) for those who experienced maternal smoking around birth were 1.19 (1.14, 1.23) for depression and 1.11 (1.07, 1.16) for anxiety. Compared with participants who experienced non-multiple births, the multivariate-adjusted HRs (95% CIs) for those who were born in multiple births were 1.16 (1.05, 1.27) for depression and 1.05 (0.95, 1.17) for anxiety. Compared with participants with normal birth weight (2.5–4 kg), the multivariate-adjusted HRs (95% CIs) for those with low birth weight (<2.5 kg) were 1.14 (1.07, 1.22) for depression and 1.12 (1.05, 1.20) for anxiety. Compared with non-adopted participants, the multivariate-adjusted HRs (95% CIs) for those adopted as children were 1.42 (1.28, 1.58) for depression and 1.25 (1.10, 1.41) for anxiety.

**Table 2:** Association of each early life factor with ICD-10 diagnosed depression and anxiety.

	*N*	Depression					Anxiety				
		Cases (%)	Model 1		Model 2		Cases (%)	Model 1		Model 2	
			HR (95% CI)	*P*_value_	HR (95% CI)	*P*_value_		HR (95% CI)	*P*_value_	HR (95% CI)	*P*_value_
Breastfed as a baby											
Yes	2,58,377	8499 (3.29)	1.00 (ref.)		1.00 (ref.)		8062 (3.12)	1.00 (ref.)		1.00 (ref.)	
No	96,851	3663 (3.78)	1.14 (1.10–1.19)	<0.001	1.08 (1.04–1.13)	<0.001	3342 (3.45)	1.10 (1.05–1.14)	<0.001	1.09 (1.04–1.13)	<0.001
Maternal smoking around birth											
No	2,84,892	9107 (3.20)	1.00 (ref.)		1.00 (ref.)		8934 (3.14)	1.00 (ref.)		1.00 (ref.)	
Yes	1,15,975	4783 (4.12)	1.30 (1.25–1.35)	<0.001	1.19 (1.14–1.23)	<0.001	4233 (3.65)	1.17 (1.13–1.21)	<0.001	1.11 (1.07–1.16)	<0.001
Part of a multiple birth											
No	4,46,413	15,624 (3.50)	1.00 (ref.)		1.00 (ref.)		14,776 (3.31)	1.00 (ref.)		1.00 (ref.)	
Yes	10,288	429 (4.17)	1.20 (1.09–1.32)	<0.001	1.16 (1.05–1.27)	0.003	369 (3.59)	1.09 (0.98–1.20)	0.119	1.05 (0.95–1.17)	0.316
Birth weight											
Normal (2.5–4 kg)	1,95,916	6483 (3.31)	1.00 (ref.)		1.00 (ref.)		6180 (3.15)	1.00 (ref.)		1.00 (ref.)	
High (>4 kg)	34,427	1229 (3.57)	1.09 (1.02–1.15)	0.008	1.04 (0.98–1.11)	0.183	1112 (3.23)	1.03 (0.97–1.10)	0.350	1.03 (0.97–1.10)	0.375
Low (<2.5 kg)	25,758	1096 (4.25)	1.30 (1.22–1.38)	<0.001	1.14 (1.07–1.22)	<0.001	1038 (4.03)	1.29 (1.21–1.38)	<0.001	1.12 (1.05–1.20)	<0.001
Adopted as a child											
No	4,56,088	16,033 (3.52)	1.00 (ref.)		1.00 (ref.)		15,125 (3.32)	1.00 (ref.)		1.00 (ref.)	
Yes	6656	346 (5.20)	1.51 (1.36–1.68)	<0.001	1.42 (1.28–1.58)	<0.001	278 (4.18)	1.28 (1.14–1.44)	<0.001	1.25 (1.10–1.41)	<0.001

Model 1 was unadjusted; Model 2 was adjusted for age, sex, ethnicity, Townsend Deprivation Index, education, current income, current smoking status, current alcohol intake, current physical activity, body mass index, and family history of depression.

*HR* hazard ratio, *CI* confidence interval.

There were no statistically significant interactions between early life risk factors on depression or anxiety (all *P* for interaction > 0.05; eTables 4–5 in the Supplement). Dose-response associations between the number of early life risk factors and the risk of incident depression and anxiety were also identified; the higher the number of early life risk factors, the higher the risk of developing depression and anxiety (all *P* for trend <0.001). Compared with the participants without any early life risk factors, those with 1, 2, and 3–4 had multivariate-adjusted HRs (95% CIs) of 1.11 (1.05, 1.17), 1.25 (1.16, 1.34), and 1.34 (1.17, 1.53) for depression, respectively; 1.10 (1.04, 1.16), 1.19 (1.10, 1.28), and 1.31 (1.14, 1.50) for anxiety, respectively (Table 3). A similar association was observed for the weighted life risk score (all *P* values for trend <0.001) (eTable 6 in the Supplement).

**Table 3:** Association of high-risk early life factors with ICD-10 diagnosed depression and anxiety.

	*N*	Depression					Anxiety				
		Cases (%)	Model 1		Model 2		Cases (%)	Model 1		Model 2	
			HR (95% CI)	*P*_trend_	HR (95% CI)	*P*_trend_		HR (95% CI)	*P*_trend_	HR (95% CI)	*P*_trend_
High-risk early life factor				<0.001		<0.001			<0.001		<0.001
0	99,272	2910 (2.93)	1.00 (ref.)		1.00 (ref.)		2866 (2.89)	1.00 (ref.)		1.00 (ref.)	
1	75,429	2636 (3.49)	1.20 (1.13–1.26)		1.11 (1.05–1.17)		2463 (3.27)	1.13 (1.07–1.20)		1.10 (1.04–1.16)	
2	26,338	1124 (4.27)	1.46 (1.36–1.56)		1.25 (1.16–1.34)		977 (3.71)	1.29 (1.20–1.38)		1.19 (1.10–1.28)	
3–4	4976	243 (4.88)	1.68 (1.47–1.92)		1.34 (1.17–1.53)		219 (4.40)	1.53 (1.34–1.76)		1.31 (1.14–1.50)	

Model 1 was unadjusted; Model 2 was adjusted for age, sex, ethnicity, Townsend Deprivation Index, education, current income, current smoking status, current alcohol intake, current physical activity, body mass index, and family history of depression.

*HR* hazard ratio, *CI* confidence interval.

### Association of early life risk factors with depression as defined by PHQ-9 and anxiety as defined by GAD-7

**Fig. 1:**
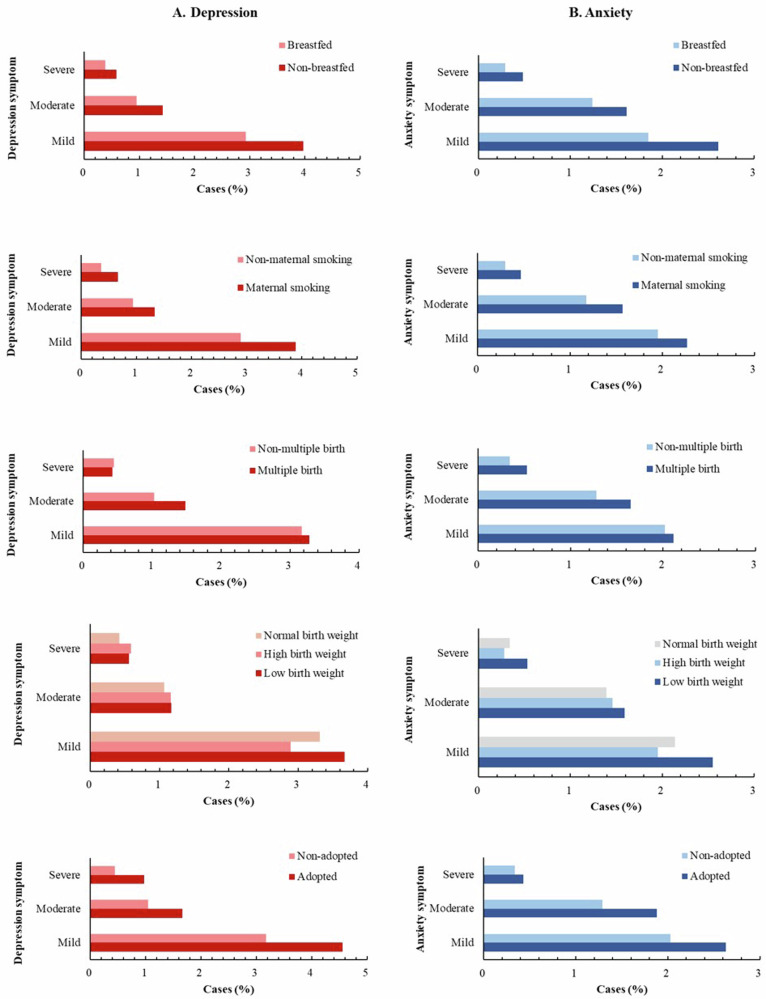
The distribution of PHQ-9 defined depression and GAD-7 defined anxiety severity according to early life factors. PHQ-9, Patient Health Questionnaire-9; GAD-7, Generalized Anxiety Disorder-7 Questionnaire; **A** depression; **B** anxiety.

In total, 1,44,561 participants completed measurements on the PHQ-9 scale, and 1,45,120 participants completed measurements on the GAD-7 scale from 2016 to 2017. After excluding individuals with known depression or anxiety at baseline, 6774 (4.69%) and 5339 (3.68%) participants were defined as having incident depression and anxiety outcomes during the follow-up period, respectively. A total of 4617 (3.19%) participants had mild depression, 1515 (1.05%) moderate depression, 642 (0.44%) severe depression, 2953 (2.03%) had mild anxiety, 1889 (1.30%) moderate anxiety, and 497 (0.34%) severe anxiety. The distribution of depression and anxiety severity as defined by the PHQ-9 and GAD-7 according to each early life factor is shown in Fig. 1 (all *P* < 0.05).

The associations of each early life risk factor with depression as defined by the PHQ-9 and anxiety as defined by the GAD-7 were shown in Table 4. Compared with participants who underwent breastfeeding, those without breastfeeding were at higher risk of developing depression and anxiety. The corresponding multivariate-adjusted ORs (95% CIs) were 1.14 (1.07, 1.21) and 1.15 (1.08, 1.23), respectively. Compared with participants whose mothers did not smoke around births, those whose mothers smoked around births were at higher risk of developing depression and anxiety. The multivariate-adjusted ORs (95% CIs) of 1.27 (1.20, 1.35) and 1.18 (1.11, 1.26), respectively. Compared with participants who experienced non-multiple births, the multivariate-adjusted ORs (95% CIs) for those who were born in multiple births were 1.14 (0.96, 1.36) for depression and 1.17 (0.97, 1.41) for anxiety. Compared with the participants with normal birth weights (2.5–4 kg), the multivariate-adjusted ORs (95% CIs) for depression and anxiety for those with low birth weights (<2.5 kg) were 1.09 (0.98, 1.22) and 1.18 (1.05, 1.33), respectively. Compared with non-adopted participants, the multivariate-adjusted ORs (95% CIs) for participants who were adopted as children were 1.43 (1.20, 1.72) for depression and 1.30 (1.05, 1.61) for anxiety.

**Table 4:** Association of each early life factor with PHQ-9 defined depression and GAD-7 defined anxiety.

	Depression					Anxiety				
	Cases (%)	Model 1		Model 2		Cases (%)	Model 1		Model 2	
		OR (95% CI)	*P*_value_	OR (95% CI)	*P*_value_		OR (95% CI)	*P*_value_	OR (95% CI)	*P*_value_
Breastfeeding										
Yes	3632 (4.25)	1.00 (ref.)		1.00 (ref.)		2896 (3.38)	1.00 (ref.)		1.00 (ref.)	
No	1804 (5.97)	1.43 (1.35–1.52)	<0.001	1.14 (1.07–1.21)	<0.001	1427 (4.70)	1.41 (1.32–1.51)	<0.001	1.15 (1.08–1.23)	<0.001
Maternal smoking around birth										
No	3788 (4.19)	1.00 (ref.)		1.00 (ref.)		3111 (3.43)	1.00 (ref.)		1.00 (ref.)	
Yes	2120 (5.89)	1.43 (1.35–1.51)	<0.001	1.27 (1.20–1.35)	<0.001	1561 (4.31)	1.27 (1.19–1.35)	<0.001	1.18 (1.11–1.26)	<0.001
Part of a multiple birth										
No	6476 (4.64)	1.00 (ref.)		1.00 (ref.)		5111 (3.64)	1.00 (ref.)		1.00 (ref.)	
Yes	147 (5.18)	1.12 (0.95–1.33)	0.173	1.14 (0.96–1.36)	0.127	122 (4.28)	1.18 (0.99–1.42)	0.072	1.17 (0.97–1.41)	0.092
Birth weight										
Normal (2.5–4 kg)	3271 (4.80)	1.00 (ref.)		1.00 (ref.)		2647 (3.87)	1.00 (ref.)		1.00 (ref.)	
High (>4 kg)	526 (4.64)	0.97 (0.88–1.06)	0.459	0.95 (0.87–1.05)	0.324	422 (3.70)	0.95 (0.86–1.06)	0.372	1.01 (0.90–1.12)	0.915
Low (<2.5 kg)	397 (5.39)	1.13 (1.02–1.26)	0.025	1.09 (0.98–1.22)	0.123	346 (4.66)	1.22 (1.08–1.36)	<0.001	1.18 (1.05–1.33)	0.005
Adopted as a child										
No	6623 (4.65)	1.00 (ref.)		1.00 (ref.)		5235 (3.66)	1.00 (ref.)		1.00 (ref.)	
Yes	134 (7.20)	1.59 (1.33–1.90)	<0.001	1.43 (1.20–1.72)	<0.001	92 (4.94)	1.37 (1.11–1.69)	<0.001	1.30 (1.05–1.61)	0.016

Model 1 was unadjusted; Model 2 was adjusted for age, sex, ethnicity, Townsend Deprivation Index, education, current income, current smoking status, current alcohol intake, current physical activity, body mass index, and family history of depression.

*OR* odds ratio, *CI* confidence interval, *PHQ-9* Patient Health Questionnaire-9, *GAD-7* Generalized Anxiety Disorder-7 Questionnaire.

## Discussion

In this large prospective cohort study, five early life factors (non-breastfeeding, maternal smoking around birth, being born in a multiple birth, low birth weight, and being adopted as a child) were identified as associated with elevated risks of incident depression and anxiety in adults aged 40–69. There were no statistically significant interactions were observed between early life risk factors on depression or anxiety. Each of these five early life factors can be considered as early life risk factors for incident depression and anxiety in adulthood independently. A dose-response association was observed, indicating that as the number of early life risk factors increased, so did the risk of experiencing depression and anxiety. These findings suggested that early life risk factors in the utero, perinatal, and early childhood development stages contribute to an increased risk of incident depression and anxiety in adulthood.

Previous studies on the relationship between early life factors and mental health support our findings. In 2012, a retrospective analysis demonstrated that a history of not being breastfed was associated with subsequent major depression in adulthood [37]. Breastfeeding was also observed as reducing the odds of developing severe depressive symptoms in adults in a birth cohort study in Brazil (*n* = 5914) [38]. Current guidelines recommend breastfeeding as a primary care intervention that can provide beneficial health outcomes across the lifespan [39–41]. The adverse effects of maternal smoking during pregnancy on birth defects and other physical diseases involved in neurodevelopment have been firmly established. Several studies have demonstrated that maternal smoking during pregnancy increases the offspring’s subsequent risk of internalizing behaviors in early childhood [22, 42]. Our findings suggest that maternal smoking around birth increases the risk of incident depression and anxiety in adulthood. The conclusions made on low birth weight were consistent with those expounded in the existing literature. A register-based study in Sweden analyzed 546,894 pairs of full siblings [27] and observed that reduced fetal growth increased the risk of developing psychiatric disorders, including depression and anxiety in adulthood. Notably, an increase of one kg in birth weight significantly decreased the risk for neurodevelopmental disorders. Similarly, two meta-analyses reported that low birth weight (<2.5 kg) is a risk factor for depression in adults [43, 44]. Studies on the relationship between being born in a multiple birth and mental diseases have rarely been reported. However, some studies have reported that individuals being born in multiple births are more likely to cause behavioral problems [17] or increase the risk of cerebral palsy [45, 46], mortality [47], and long-term disease [48–50]. In this large sample study, being born in multiple births was observed as being associated with significantly higher risks of depression and anxiety in adulthood as compared to not being born in multiple births, supporting the notion that being born in multiple births is a risk factor for mental illness. Given the widening application of medically assisted conception, the corresponding multiple pregnancy rate is also increasing [51], with the mental health problems associated with multiple births subsequently requiring more attention. The mental health of adopted children is always a concern, with some studies having reported that adoptees are at a high risk of developing general behavioral problems during adolescence [16, 52, 53]. Westermeyer et al. investigated the lifelong influence of childhood adoption [15] and found that adoptees (*n* = 378) had increased lifetime odds ratios of developing major depression, generalized anxiety disorder, and other internalized mental disorders from adolescence to middle and old age, as compared to non-adoptees (*n* = 42,503). Taken together with our findings, adoption in early childhood can be clearly stated as having a long-term impact on mental health in adulthood.

Several mechanisms have been proposed to explain the association between these early life risk factors and subsequent mental health issues. Firstly, these early life risk factors directly affect fetal brain neurodevelopment and physiological systems related to emotional regulation [54–58]. For instance, prenatal nicotine exposure directly stimulate fetal nicotinic acetylcholine receptors, affecting neurotransmitter systems in brain regions like the hippocampus and somatosensory cortex [55, 59]; low birth weights are related to intrauterine growth retardation; and the protective role of breastfeeding on mental health including the antidepressant biological components of breast milk, such as oxytocin [58] and docosahexaenoic acid [60].

Secondly, early social environments and relationships, such as early attachment and psychological stressors, significantly influence children’s psychological development and emotional regulation. The childhood adversity often faced by adopted children, such as issues of identity, unknown origin, and discrimination; multiple births grow up in a more competitive environment, mostly suffering the preferences of parent [50] and the increased risk of child abuse in families [61]; breastfeeding were also reported being associated with improved mother-infant bonding [62, 63]. Furthermore, epigenetic studies have shown that environmental exposures, like maternal smoking or nutritional deficiencies, can alter the methylation patterns of genes, affecting the manifestation of psychological and behavioral traits [64–67].

This study holds significant public health implications for the early identification of risk factors associated with depression and anxiety in adulthood. The status of related risk factors in early life affects the risk of certain diseases, including cardiovascular diseases and mental disorders in adults [18, 19, 68, 69]. Our findings supported the suggestion that the five identified early life risk factors increase the risk of mental health problems developing in middle-aged and older adults. This suggested that strategies for the prevention of depression and anxiety in adulthood should focus heavily on the early stages of life, and interventions targeting these five early life risk factors should be incorporated into primary health care.

The present study has some limitations. First, the collected information was based on individuals’ recall of their early life when they were adults, which may have been subject to recall bias. Second, some residual confounders may have influenced our results, although the main covariates were adjusted for in the regression models. Finally, the study population included in this study aged 40–69 years; thus, the findings may not generalize to all age groups. Excluding individuals with pre-existing depression or anxiety at baseline may have contributed to the low prevalence of depression/anxiety in this study.

## Conclusion

In this study, early life risk factors, including non-breastfeeding, maternal smoking around birth, being born in multiple births, low birth weight, and being an adoptee were found to elevate the risks of developing depression and anxiety in adulthood. These findings emphasize the importance of directing prevention strategies for depression and anxiety in adulthood toward the early stages of life.

### Supplementary information

Supplementary Online Content

## Data Availability

All data generated or analyzed during this study are included in this publication and/or are available from the corresponding author on reasonable request.

## Code availability

Codes used in the current study are available from the corresponding author upon reasonable request.
