# Supplementary material for: The association of early life factors with depression and anxiety in adults aged 40–69 years: a population-based cohort study
Source: Transl Psychiatry. 2024 Jul 20;14:299. doi: 10.1038/s41398-024-03006-7 (PMC11271466; doi:10.1038/s41398-024-03006-7)
Supplement: Supplementary file 1 — Supplementary Online Content [file 41398_2024_3006_MOESM1_ESM.docx]

**Supplementary Online Content**

**The association of early life factors with depression and anxiety in adults aged 40–69 years:**

**a population-based cohort study**

Ruirui Wang, MD; Mengyao Shi, MD, PhD; Qilu Zhang, MD; Jing Zhang, MS; Lulu Sun, MD; Yiming Jia, MD; Zhengbao Zhu, MD, PhD; Tan Xu, MD, PhD; Yonghong Zhang, MD, PhD

**eMethods.** Statistical analysis

**eTable 1.** Patient Health Questionnaire-9 (PHQ-9) scale

**eTable 2.** Generalized Anxiety Disorder-7 Questionnaire (GAD-7) scale

**eTable 3**. Baseline characteristics of the population included in the main analyses and secondary analyses

**eTable 4.** Hazard ratios and 95% confidence intervals of each early life factor with ICD-10 diagnosed depression in different subgroup

**eTable 5.** Hazard ratios and 95% confidence intervals of each early life factor with ICD-10 diagnosed anxiety in different subgroup

**eTable 6.** Association of weighted high-risk early life factors with ICD-10 diagnosed depression and anxiety

**eFigure 1.** Flow diagram of participant selection

**eMethods. Statistical analysis**

We constructed a weighted high-risk early life score based on the four early life factors, including breastfeeding, maternal smoking around birth, multiple births, and birth weight. Each early life factor was coded 1 if meeting the high-risk criterion and 0 if not. High-risk early life factors were defined as non-breastfeeding, maternal smoking around birth, being a part of multiple birth, and low birth weight. Using the equation: weighted high-risk early life score = (β1 × factor 1 + β2 × factor 2 + β3 × factor 3 + β4 × factor 4) × (4/sum of the β coefficients)^1,2^. Here, β1, β2, β3, and β4 denoted the estimates of β coefficients for breastfeeding, maternal smoking around birth, multiple birth, and birth weight, respectively, which were obtained by fitting the multivariable Cox proportional hazards models for depression and anxiety.

**eTable 1. Patient Health Questionnaire-9 (PHQ-9) scale**

| **Field ID** | **Question:** Over the last 2 weeks, how often have you been bothered by any of the following problems? | **Option:** Select one from the following for each of the statements |
| --- | --- | --- |
| 20514 | Little interest or pleasure in doing things | - 01 Not at all  - 02 Several days  - 03 More than half the days  - 04 Nearly every day  - DA Prefer not to answer |
| 20510 | Feeling down, depressed, or hopeless |  |
| 20517 | Trouble falling or staying asleep, or sleeping too much |  |
| 20519 | Feeling tired or having little energy |  |
| 20511 | Poor appetite or overeating |  |
| 20507 | Feeling bad about yourself or that you are a failure or have let yourself or your family down |  |
| 20508 | Trouble concentrating on things, such as reading the newspaper or watching television |  |
| 20518 | Moving or speaking so slowly that other people could have noticed? Or the opposite — being so fidgety or restless that you have been moving around a lot more than usual |  |
| 20513 | Thoughts that you would be better off dead or of hurting yourself in some way |  |

**eTable 2. Generalized Anxiety Disorder-7 Questionnaire (GAD-7) scale**

| **Field ID** | **Question:** Over the last 2 weeks, how often have you been bothered by any of the following problems? | **Option:** Select one from the following for each of the statements |
| --- | --- | --- |
| 20506 | Feeling nervous, anxious or on edge | - 01 Not at all  - 02 Several days  - 03 More than half the days  - 04 Nearly every day  - DA Prefer not to answer |
| 20509 | Not being able to stop or control worrying |  |
| 20520 | Worrying too much about different things |  |
| 20515 | Trouble relaxing |  |
| 20516 | Being so restless that it is hard to sit still |  |
| 20505 | Becoming easily annoyed or irritable |  |
| 20512 | Feeling afraid as if something awful might happen |  |

**eTable 3. Baseline characteristics of the population included in the main analyses and secondary analyses**

| **Baseline characteristics** | **Individuals included in the main analysis** | **Individuals completed the online follow-up of depression scale (PHQ-9)** | **Individuals completed online follow-up of anxiety scale (GAD-7)** |
| --- | --- | --- | --- |
| Number of participants | 502394 | 154312 | 155027 |
| Age, years | 56.53±8.10 | 55.90±7.74 | 55.89±7.73 |
| Male, % | 229079 (45.60) | 67134 (43.51) | 67452 (43.51) |
| White, % | 475135 (94.57) | 149,891 (97.14) | 150577 (97.13) |
| Townsend Deprivation Index | -1.29±3.10 | -1.71±2.83 | -1.71±2.83 |
| University or college degree, % | 164241 (32.69) | 70496 (45.68) | 70744 (45.63) |
| Low income, % | 118763 (23.64) | 22377 (14.50) | 22573 (14.56) |
| Current cigarette smoking, % | 53344 (10.62) | 11121 (7.21) | 11218 (7.24) |
| Current alcohol drinking, % | 461704 (91.90) | 145767 (94.46) | 146411 (94.44) |
| Low physical activity, % | 180146 (35.86) | 47585 (30.84) | 47918 (30.91) |
| Body mass index, kg/m^2^ | 27.43±4.80 | 26.77±4.55 | 26.78±4.56 |
| Family history of depression, % | 45616 (9.08) | 15640 (10.14) | 15770 (10.17) |
| Early life factor, % |  |  |  |
| Breastfed as a baby | 277533 (72.35) | 90866 (73.61) | 91259 (73.58) |
| Maternal smoking around birth | 126590 (29.25) | 38766 (28.73) | 39009 (28.78) |
| Part of a multiple birth | 11230 (2.28) | 3056 (2.01) | 3066 (2.01) |
| Birth weight |  |  |  |
| Normal (2.5-4 kg) | 211589 (76.41) | 72922 (78.48) | 73239 (78.41) |
| High (>4 kg) | 37111 (13.40) | 12083 (13.00) | 12177 (13.04) |
| low (<2.5 kg) | 28227 (10.19) | 7918 (8.52) | 7985 (8.55) |
| Adopted as a child | 7353 (1.47) | 2007 (1.30) | 2014 (1.30) |

Continuous variables are expressed as mean ± standard deviation; categorical variables are expressed as number (percentage). PHQ-9, Patient Health Questionnaire-9; GAD-7, Generalized Anxiety Disorder-7 Questionnaire.

**eTable 4. Hazard ratios and 95% confidence intervals of each early life factor with ICD-10 diagnosed depression in different subgroup**

|  | **Model 1** |  |  | **Model 2** |  |  |
| --- | --- | --- | --- | --- | --- | --- |
|  | **Non-breastfeeding** | **Breastfeeding** | ***P* _interaction_** | **Non-breastfeeding** | **Breastfeeding** | ***P* _interaction_** |
| **Maternal smoking around birth** |  |  | 0.709 |  |  | 0.677 |
| No | 1.10 (1.04-1.16) | 1.00 (ref) |  | 1.07 (1.02-1.13) | 1.00 (ref) |  |
| Yes | 1.12 (1.04-1.19) | 1.00 (ref) |  | 1.02 (0.95-1.09) | 1.00 (ref) |  |
| **Part of a multiple birth** |  |  | 0.541 |  |  | 0.587 |
| No | 1.13 (1.09-1.18) | 1.00 (ref) |  | 1.07 (1.03-1.12) | 1.00 (ref) |  |
| Yes | 1.06 (0.85-1.32) | 1.00 (ref) |  | 0.96 (0.76-1.20) | 1.00 (ref) |  |
| **Birth weight** |  |  | 0.053 |  |  | 0.056 |
| Normal (2.5-4 kg) | 1.10 (1.04-1.16) | 1.00 (ref) |  | 1.03 (0.97-1.09) | 1.00 (ref) |  |
| High (>4 kg) | 1.05 (0.92-1.21) | 1.00 (ref) |  | 1.03 (0.90-1.19) | 1.00 (ref) |  |
| Low (<2.5 kg) | 1.29 (1.13-1.47) | 1.00 (ref) |  | 1.20 (1.05-1.37) | 1.00 (ref) |  |
| **Adopted as a child** |  |  | 0.606 |  |  | 0.562 |
| No | 1.13 (1.09-1.18) | 1.00 (ref) |  | 1.07 (1.03-1.11) | 1.00 (ref) |  |
| Yes | 1.03 (0.73-1.47) | 1.00 (ref) |  | 1.23 (0.85-1.78) | 1.00 (ref) |  |
|  | **Non-maternal smoking** | **Maternal smoking** | ***P* _interaction_** | **Non-maternal smoking** | **Maternal smoking** | ***P* _interaction_** |
| **Part of a multiple birth** |  |  | 0.689 |  |  | 0.705 |
| No | 1.00 (ref) | 1.30 (1.26-1.35) |  | 1.00 (ref) | 1.19 (1.15-1.23) |  |
| Yes | 1.00 (ref) | 1.24 (1.00-1.54) |  | 1.00 (ref) | 1.11 (0.89-1.39) |  |
| **Birth weight** |  |  | 0.398 |  |  | 0.389 |
| Normal (2.5-4 kg) | 1.00 (ref) | 1.34 (1.27-1.41) |  | 1.00 (ref) | 1.19 (1.13-1.26) |  |
| High (>4 kg) | 1.00 (ref) | 1.30 (1.14-1.48) |  | 1.00 (ref) | 1.23 (1.08-1.40) |  |
| Low (<2.5 kg) | 1.00 (ref) | 1.25 (1.10-1.43) |  | 1.00 (ref) | 1.11 (0.97-1.26) |  |
| **Adopted as a child** |  |  | - |  |  | - |
| No | 1.30 (1.25-1.34) | 1.00 (ref) |  | 1.18 (1.14-1.23) | 1.00 (ref) |  |
| Yes | - | - |  | - | - |  |
|  | **Non-multiple birth** | **Multiple birth** | ***P* _interaction_** | **Non-multiple birth** | **Multiple birth** | ***P* _interaction_** |
| **Birth weight** |  |  | 0.052 |  |  | 0.191 |
| Normal (2.5-4 kg) | 1.00 (ref) | 1.27 (1.06-1.52) |  | 1.00 (ref) | 1.26 (1.04-1.52) |  |
| High (>4 kg) | 1.00 (ref) | 0.49 (0.12-1.95) |  | 1.00 (ref) | 0.46 (0.12-1.84) |  |
| Low (<2.5 kg) | 1.00 (ref) | 0.97 (0.82-1.15) |  | 1.00 (ref) | 1.04 (0.88-1.23) |  |
| **Adopted as a child** |  |  | - |  |  | - |
| No | 1.20 (1.09-1.32) | 1.00 (ref) |  | 1.16 (1.05-1.28) | 1.00 (ref) |  |
| Yes | - | - |  | - | - |  |
|  | **Non-adopted** | **Adopted** | ***P* _interaction_** | **Adopted** | **Non-adopted** | ***P* _interaction_** |
| **Birth weight** |  |  | 0.375 |  |  | 0.422 |
| Normal (2.5-4 kg) | 1.00 (ref) | 1.27 (1.06-1.52) |  | 1.00 (ref) | 1.23 (1.03-1.47) |  |
| High (>4 kg) | 1.00 (ref) | 0.49 (0.12-1.97) |  | 1.00 (ref) | 0.47 (0.12-1.87) |  |
| Low (<2.5 kg) | 1.00 (ref) | 0.97 (0.82-1.15) |  | 1.00 (ref) | 1.04 (0.88-1.23) |  |

Model 1 was unadjusted; Model 2 was adjusted for age, sex, ethnicity, Townsend Deprivation Index, education, current income, current smoking status, current alcohol intake, current physical activity, body mass index, and family history of depression.

**eTable 5. Hazard ratios and 95% confidence intervals of each early life factor with ICD-10 diagnosed anxiety in different subgroup**

|  | **Model 1** |  |  | **Model 2** |  |  |
| --- | --- | --- | --- | --- | --- | --- |
|  | **Non-breastfeeding** | **Breastfeeding** | ***P* _interaction_** | **Non-breastfeeding** | **Breastfeeding** | ***P* _interaction_** |
| **Maternal smoking around birth** |  |  | 0.607 |  |  | 0.793 |
| No | 1.07 (1.01-1.13) | 1.00 (ref) |  | 1.09 (1.03-1.15) | 1.00 (ref) |  |
| Yes | 1.09 (1.02-1.18) | 1.00 (ref) |  | 1.06 (0.98-1.14) | 1.00 (ref) |  |
| **Part of a multiple birth** |  |  | 0.868 |  |  | 0.830 |
| No | 1.09 (1.05-1.14) | 1.00 (ref) |  | 1.08 (1.04-1.13) | 1.00 (ref) |  |
| Yes | 1.07 (0.85-1.35) | 1.00 (ref) |  | 1.04 (0.82-1.32) | 1.00 (ref) |  |
| **Birth weight** |  |  | 0.969 |  |  | 0.846 |
| Normal (2.5-4 kg) | 1.07 (1.01-1.13) | 1.00 (ref) |  | 1.07 (1.01-1.14) | 1.00 (ref) |  |
| High (>4 kg) | 1.00 (0.86-1.15) | 1.00 (ref) |  | 1.01 (0.87-1.17) | 1.00 (ref) |  |
| Low (<2.5 kg) | 1.07 (0.94-1.23) | 1.00 (ref) |  | 1.06 (0.92-1.22) | 1.00 (ref) |  |
| **Adopted as a child** |  |  | 0.624 |  |  | 0.910 |
| No | 1.09 (1.05-1.14) | 1.00 (ref) |  | 1.08 (1.04-1.13) | 1.00 (ref) |  |
| Yes | 0.99 (0.67-1.46) | 1.00 (ref) |  | 1.06 (0.70-1.59) | 1.00 (ref) |  |
|  | **Non-maternal smoking** | **Maternal smoking** | ***P* _interaction_** | **Non-maternal smoking** | **Maternal smoking** | ***P* _interaction_** |
| **Part of a multiple birth** |  |  | 0.876 |  |  | 0.745 |
| No | 1.00 (ref) | 1.17 (1.13-1.21) |  | 1.00 (ref) | 1.11 (1.07-1.16) |  |
| Yes | 1.00 (ref) | 1.19 (0.94-1.51) |  | 1.00 (ref) | 1.18 (0.93-1.50) |  |
| **Birth weight** |  |  | 0.577 |  |  | 0.596 |
| Normal (2.5-4 kg) | 1.00 (ref) | 1.21 (1.14-1.28) |  | 1.00 (ref) | 1.14 (1.07-1.21) |  |
| High (>4 kg) | 1.00 (ref) | 1.18 (1.03-1.35) |  | 1.00 (ref) | 1.14 (0.99-1.31) |  |
| Low (<2.5 kg) | 1.00 (ref) | 1.15 (1.00-1.31) |  | 1.00 (ref) | 1.08 (0.95-1.24) |  |
| **Adopted as a child** |  |  | - |  |  | - |
| No | 1.00 (ref) | 1.17 (1.13-1.21) |  | 1.00 (ref) | 1.11 (1.07-1.16) |  |
| Yes | 1.00 (ref) | - |  | 1.00 (ref) |  |  |
|  | **Non-multiple birth** | **Multiple birth** | ***P* _interaction_** | **Non-multiple birth** | **Multiple birth** | ***P* _interaction_** |
| **Birth weight** |  |  | 0.791 |  |  | 0.946 |
| Normal (2.5-4 kg) | 1.00 (ref) | 1.12 (0.92-1.36) |  | 1.00 (ref) | 1.11 (0.92-1.35) |  |
| High (>4 kg) | 1.00 (ref) | 0.54 (0.13-2.15) |  | 1.00 (ref) | 0.52 (0.13-2.08) |  |
| Low (<2.5 kg) | 1.00 (ref) | 1.04 (0.88-1.23) |  | 1.00 (ref) | 1.09 (0.92-1.29) |  |
| **Adopted as a child** |  |  | - |  |  | - |
| No | 1.00 (ref) | 1.09 (0.98-1.20) |  | 1.00 (ref) | 1.06 (0.95-1.17) |  |
| Yes | 1.00 (ref) | - |  | 1.00 (ref) | - |  |
|  | **Non-adopted** | **Adopted** | ***P* _interaction_** | **Adopted** | **Non-adopted** | ***P* _interaction_** |
| **Birth weight** |  |  | 0.334 |  |  | 0.333 |
| Normal (2.5-4 kg) | 1.00 (ref) | 1.12 (0.92-1.36) |  | 1.00 (ref) | 1.11 (0.92-1.35) |  |
| High (>4 kg) | 1.00 (ref) | 0.54 (0.14-2.17) |  | 1.00 (ref) | 0.53 (0.13-2.11) |  |
| Low (<2.5 kg) | 1.00 (ref) | 1.04 (0.88-1.23) |  | 1.00 (ref) | 1.09 (0.92-1.29) |  |

Model 1 was unadjusted; Model 2 was adjusted for age, sex, ethnicity, Townsend Deprivation Index, education, current income, current smoking status, current alcohol intake, current physical activity, body mass index, and family history of depression.

**eTable 6. Association of weighted high-risk early life factors with ICD-10 diagnosed depression and anxiety**

|  | **Depression** | | | | | **Anxiety** | | | | |
| --- | --- | --- | --- | --- | --- | --- | --- | --- | --- | --- |
|  | **Cases (%)** | **Model 1** |  | **Model 2** |  | **Cases (%)** | **Model 1** |  | **Model 2** |  |
|  |  | **HR (95% CI)** | ***P* _trend_** | **HR (95% CI)** | ***P* _trend_** |  | **HR (95% CI)** | ***P* _trend_** | **HR (95% CI)** | ***P* _trend_** |
| **High-risk early life score** |  |  | <0.001 |  | <0.001 |  |  | <0.001 |  | <0.001 |
| **Q1** | 2910 (2.93) | 1.00 (ref) |  | 1.00 (ref) |  | 2866 (2.89) | 1.00 (ref) |  | 1.00 (ref) |  |
| **Q2** | 1085 (3.13) | 1.06 (0.99-1.14) |  | 1.01 (0.94 -1.09) |  | 1328 (3.13) | 1.08 (1.01-1.16) |  | 1.06 (0.99-1.13) |  |
| **Q3** | 1551 (3.81) | 1.31 (1.23-1.39) |  | 1.15 (1.08-1.22) |  | 1171 (3.43) | 1.20 (1.12-1.28) |  | 1.10 (1.03-1.18) |  |
| **Q4** | 1367 (4.37) | 1.50 (1.40-1.60) |  | 1.20 (1.12-1.28) |  | 1160 (3.83) | 1.33 (1.24-1.42) |  | 1.16 (1.08-1.24) |  |

HR, hazard ratio; CI, confidence interval; Model 1 was unadjusted; Model 2 was adjusted for age, sex, ethnicity, Townsend Deprivation Index, education, current income, current smoking status, current alcohol intake, current physical activity, body mass index, and family history of depression.

**eFigure 1. Flow diagram of participant selection**

**
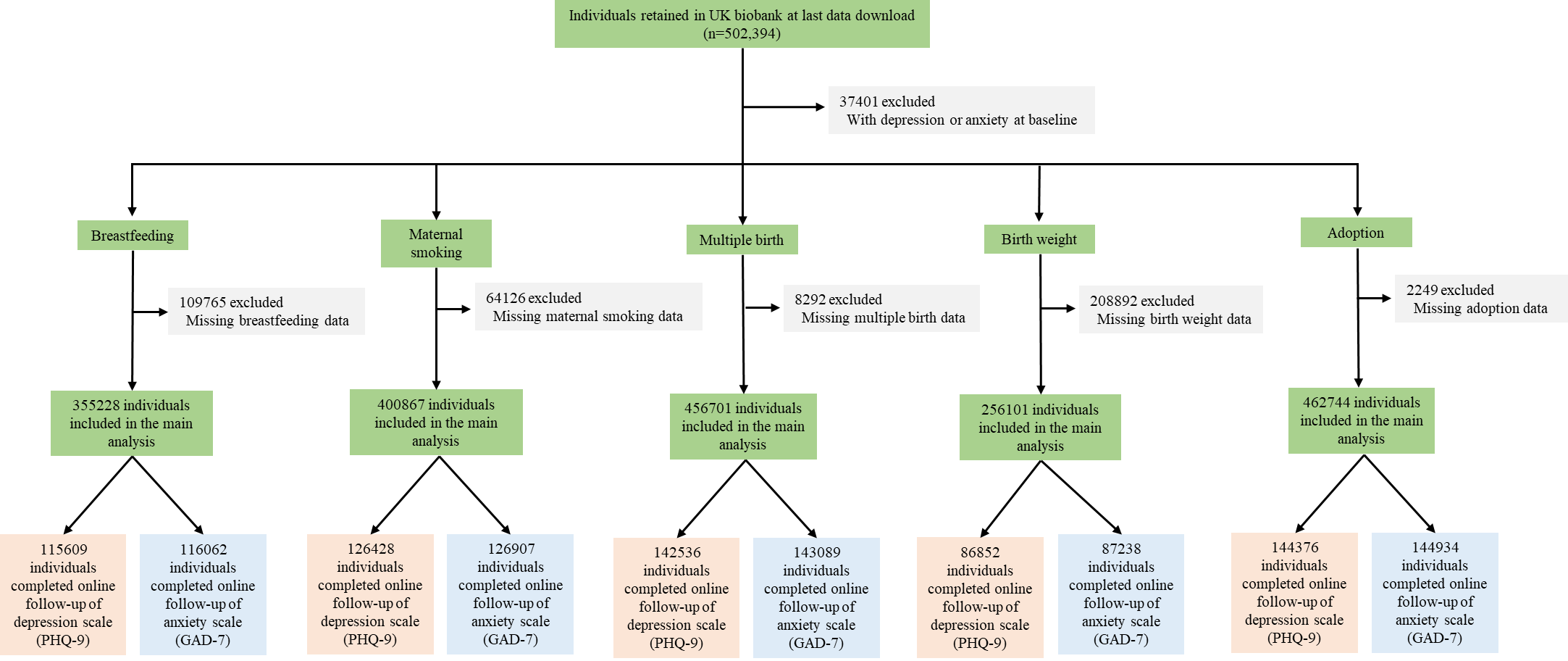
**

**eReferences**

1. Fan M, Sun D, Zhou T, et al. Sleep patterns, genetic susceptibility, and incident cardiovascular disease: a prospective study of 385 292 UK biobank participants. *Eur Heart J.* 2020;41(11):1182-1189.

2. Khera AV, Emdin CA, Drake I, et al. Genetic Risk, Adherence to a Healthy Lifestyle, and Coronary Disease. *N Engl J Med.* 2016;375(24):2349-2358.
